# A novel mutation in the mouse *Pcsk1* gene showing obesity and diabetes

**DOI:** 10.1007/s00335-020-09826-4

**Published:** 2020-01-23

**Authors:** Nor I. A. Muhsin, Liz Bentley, Ying Bai, Michelle Goldsworthy, Roger D. Cox

**Affiliations:** Mammalian Genetics Unit, MRC Harwell Institute, Harwell Campus, Oxfordshire, OX11 0RD UK

**Keywords:** Proprotein convertase subtilisin/Kexin type 1 (PCSK1/PC1), Obesity, Hyperphagia, Proinsulin, Diabetes

## Abstract

The proprotein convertase subtilisin/Kexin type 1 (PCSK1/PC1) protein processes inactive pro-hormone precursors into biologically active hormones in a number of neuroendocrine and endocrine cell types. Patients with recessive mutations in *PCSK1* exhibit a complex spectrum of traits including obesity, diarrhoea and endocrine disorders. We describe here a new mouse model with a point mutation in the *Pcsk1* gene that exhibits obesity, hyperphagia, transient diarrhoea and hyperproinsulinaemia, phenotypes consistent with human patient traits. The mutation results in a pV96L amino acid substitution and changes the first nucleotide of mouse exon 3 leading to skipping of that exon and in homozygotes very little full-length transcript. Overexpression of the exon 3 deleted protein or the 96L protein results in ER retention in Neuro2a cells. This is the second *Pcsk1* mouse model to display obesity phenotypes, contrasting knockout mouse alleles. This model will be useful in investigating the basis of endocrine disease resulting from prohormone processing defects.

## Introduction

Proprotein convertases (PCs) proteolytically processes inactive pro-hormone precursors into biologically active peptides (Turpeinen et al. 2013). Proprotein convertase subtilisin/Kexin types 1 and 2 (PCSK1 and PCSK2) are found in neuroendocrine and endocrine cells cleaving a range of targets including proinsulin (Furuta et al. 1998; Smeekens et al. 1992; Zhu et al. 2002a), progonadotrophin-releasing hormone (GnRH) (Wetsel et al. 1995), proopiomelanocortin (POMC)(Benjannet et al. 1991; Zhou et al. 1993), proglucagon (Dhanvantari et al. 1996; Rouille et al. 1995), prothyrotrophin-releasing hormone (Schaner et al. 1997), pro-growth hormone-releasing hormone (pro-GHRH) (Dey et al. 2004; Posner et al. 2004), proghrelin (Zhu et al. 2006) and prosomatostatin (Galanopoulou et al. 1993).

At least 26 cases of congenital recessive PCSK1 deficiency have been reported, and consistent with their function exhibited a variable range of symptoms that include malabsorptive diarrhoea, obesity and various endocrine disorders (reviewed Pepin et al. 2019; Stijnen et al. 2016). These latter including hyperproinsulinaemia, hypogonadism, hypercortisolism, postprandial hypoglycaemia, hypothyroidism and growth hormone (GH) deficiency (Pepin et al. 2019; Stijnen et al. 2016). Dominantly inherited rare mutations impacting on obesity have also been described suggesting dominant negative effects (Creemers et al. 2012; Philippe et al. 2015; Stijnen et al. 2016). Single-nucleotide polymorphisms in genome wide association studies have linked the *PCSK1* locus with obesity demonstrating a role for the locus in susceptibility to commonly occurring obesity in the population (Benzinou et al. 2008; Nead et al. 2015; Stijnen et al. 2014) and with fasting proinsulin (Heni et al. 2010; Strawbridge et al. 2011). Finally, it has been reported that deficiency of PCSK1 impairs prohormone processing in Prader-Willi syndrome (Burnett et al. 2017).

An exon 1 deletion in the mouse resulted in growth retardation, with mice being about 60% of normal size at 10 weeks due to low pituitary GH as a result of a GHRH maturation defect (Zhu et al. 2002b). These homozygous mice are not obese (heterozygotes were mildly obese) and do not show impairment of glucose tolerance (heterozygotes were mildly glucose intolerant possibly as a result of their mild obesity) although they have POMC processing defects, hyperproinsulinaemia and a block on intestinal glucagon like peptide-1 and -2 production (Zhu et al. 2002b). These mice have also been reported to have defects in macrophage cytokine secretion (Refaie et al. 2012). In a second mouse, model exons 3 to 9 were deleted resulting in preimplantation lethality in homozygotes (Mbikay et al. 2007).

A mouse model, with a point mutation causing a N222D amino acid substitution, that better phenocopies human PCSK1 deficiency was reported by Lloyd et al*.* This model exhibits obesity, possibly due to reduced POMC processing and consequently lower levels of anorexic alpha-MSH hormone (Lloyd et al. 2006). Further, these mice were not growth retarded and showed normal pro-GHRH processing (Lloyd et al. 2006). Glucose intolerance as a consequence of abnormal proinsulin processing was also observed (Lloyd et al. 2006).

Additional mouse models that better replicate the human traits, in addition to N222D, would be of value in further understanding the human genetic variation that gives rise to obesity and metabolic disease. We have identified a new *Pcsk1* mouse mutant in a *N-ethyl-N-nitrosourea* (ENU) mutagenesis screen for age related disease (Potter et al. 2016). These mice in addition to a pV96L missense change also showed mis-splicing of exon 3 and exhibited obesity, hyperphagia, glucose intolerance, insulin resistance, hyperproinsulinaemia and transient diarrhoea. Both the 96L and exon 3 deletion proteins were colocalised to some degree to the ER indicating defective transport.

## Materials and methods

### Animal models

All mice were housed in the Mary Lyon Centre at MRC Harwell in accordance with UK Home Office legislation and local ethical guidelines issued by the Medical Research Council (Responsibility in the Use of Animals for Medical Research, July 1993; Home Office licence 30/3146 and 30/3070). Housing was under controlled light (light 7 a.m.–7 p.m., dark 7 p.m.–7 a.m.), temperature (21 ± 2 °C) and humidity (55 ± 10%) conditions. Mice had free access to water (9–13 ppm chlorine) and were fed ad libitum on a commercial diet (Special Diets Service (SDS) rat and mouse no. 3 breeding diet, RM3, 3.6 kcal/g).

### Body weight and composition analysis

Body composition was determined using an Echo-MRI quantitative NMR machine (Echo-MRI-100, Echo-MRI, Texas, USA). Body mass was measured using scales calibrated to 0.01 g.

### Food intake

Food intake was measured according to Moir et al. (2016). Briefly, mice were housed in pairs of the same sex and genotype and each individual cage given a known amount of diet which was re-weighed daily between 9 a.m. and 10 a.m. on a sensitive balance accurate to three decimal places (Ohaus Explorer Pro, Ohaus Europe GmbH, Switzerland). Food was topped up to 80 g each day. Daily food intake for each mouse was calculated by dividing the cage value by 2.

### Glucose tolerance tests

Intraperitoneal glucose tolerance tests (IPGTT) were performed in the morning after an overnight fast (up to 18 h). The mice were weighed and an approximately 100 μl blood sample (*T *= 0 glucose baseline) was collected from the lateral tail vein into CB300 lithium-heparin microvette tubes (Sarstedt, Leicester, UK) for insulin concentration measurement. Blood was then centrifuged at 4 °C at 3000 rpm for 10 min to collect the plasma and stored at − 20 °C until use. Blood glucose concentrations were measured immediately using AlphaTrak2 glucose meter (Abbott Laboratories, Illinois, USA). Subsequent blood glucose was measured at 60 and 120 min using AlphaTrak2 glucose metre (Abbott Laboratories, Illinois, USA) after receiving intraperitoneal injection of 2 g glucose/kg body weight (20% glucose in 0.9% Sodium Chloride (NaCl) filter sterilized and stored at − 20 °C until use). Food was returned at the end of the procedure.

### Insulin and proinsulin ELISA

Plasma samples were collected and Insulin and Proinsulin ELISA assays (Mercodia 10-1247-01 and 10-1232-01, respectively) carried out according to the manufacturer’s instructions. According to the manufacturer, the proinsulin assay is specific for proinsulin and the insulin assay detects both insulin and proinsulin I (43%) and proinsulin II (60%).

### SNP mapping and whole genome sequencing

SNP mapping and NGS were performed as described previously (Potter et al. 2016). Briefly Individual mutations were mapped using the Illumina GoldenGate Mouse Medium Density Linkage Panel (Gen-Probe Life Sciences Ltd, UK) that utilizes over 900 single-nucleotide polymorphisms (SNPs) for the C3H/Pde (Pde6b+ repaired mice) and C57BL/6J strains.

### Quantitative PCR analysis

Total pancreatic RNA was extracted using a Precellys soft tissue homogenizing CK14 kit (VWR International, Pennsylvania, USA) and a RNeasy Plus mini-kit (Qiagen) according to the manufacturer’s instructions. RNA concentration was determined using an Epoch spectrophotometer (BioTek) using a Take3 Micro-Volume Plates (BioTek). RNA quality was assessed using the Bioanalyser with the RNA 6000 Nano Kit (Agilent Technologies, California, USA). RNA was stored at 80 °C until use.

Complementary DNA (cDNA) was generated using high-quality RNA template by reverse transcriptase using a Maxima First Strand cDNA Synthesis Kit for qRT-PCR (Thermo Fisher Scientific Inc., Massachusetts, USA). Reactions were set up according to manufacturer’s instructions. cDNA was stored at − 20 °C.

cDNA samples were analysed by Quantitative Real-Time PCR (qRT-PCR) using TaqMan^®^ probes with FAM tags based on real-time detection of accumulated fluorescence (ABI Prism 7500, Applied Biosystems, California, USA). A custom Taqman probe for the *Pcsk1* Mutant was designed and the forward primer sequence was CTCGGAGGTCCCGAAGAAG, the reverse primer sequence was GGCAGAGCTGCAGTCATTCTG and the probe sequence was TGATGATCGTCAAGATA. The wildtype *Pcsk1* assay and the endogeneous control *Hprt1* probes, Mm01345252_g1 and Mm03024075_m1, respectively, were purchased from Applied Biosystems.

qRT-PCR reactions were set up in triplicate using 10 ng of pre-amplified cDNA per well. Reactions were performed using a standard protocol of 95 °C for 20 s to activate Taq polymerase, followed by 40 cycles of denaturation at 95 °C for 1 s and annealing and extension at 60 °C for 20 s. Results were analysed with the ExpressionSuite software (Life Technologies, California, USA) using the averaged expression values of *Hprt1* as the endogenous control.

### Expression of tagged proteins

A mammalian gene collection (MGC) Fully Sequenced Mouse *Pcsk1* cDNA (accession number: BC108982) in pCR-BluntII TOPO vector was obtained from Dharmacon GE Healthcare Life Sciences, Buckinghamshire, UK. This cDNA was sequence verified. Site-directed mutagenesis was carried out using a Quikchange^®^ Multi Site-Directed Mutagenesis Kit (Agilent Technologies, California, USA). We firstly introduced a SacII restriction site at the 3′ end of the cDNA to remove the stop codon and to enable subsequent fusion of myc tag epitope. Two additional C-terminal modifications were made to generate an uncleavable C-terminal end of PCSK1 protein in order to prevent cleavage of the cMYC C-terminal tag (Bernard et al. 2003). This template was then used to generate 2 additional cDNA constructs one containing the V96L point mutation (PCSK1-V96L), and one the exon 3 deletion (PCSK1-Deletion). Mutated *Pcsk1* cDNAs were then inserted into a pcDNA^TM^3.1/mycB vector, containing cytomegalovirus (CMV) promoter with a C-terminal myc tag.

Transfection of plamsids into Neuro2a cells grown on coverslips was carried out using Lipofectamine 3000 (Invitrogen) according to the manufacturer’s instructions. After 48 h cells were fixed in 4% paraformaldehyde (PFA) at room temperature for 20 min and then permeabilised with 0.1% Triton-X-100 (Sigma-Aldrich) for 10 min at room temperature. Cells were then incubated with primary antibodies [Mouse Anti-c-Myc, diluted 1:250 (Invitrogen, Catalogue number 132500), Rabbit Anti-PDI (protein disulphide isomerase), diluted 1:250 (Cell Signalling Technology); Rabbit Anti-RCAS1 (receptor binding cancer antigen expressed on SiSo cells) 1:250 (CST, Catalogue number 12290); Rabbit anti-POMC (proopiomelanocortin) diluted 1:250 (Antibodies-online, Catalogue number ABIN1077682) in blocking buffer (phosphate buffered saline (PBS) with 5% donkey serum (Sigma-Aldrich, D9663)] at 4 °C overnight. The next day, cells were washed with PBS with 0.1% Tween20 (PBST) three times and then incubated with Invitrogen Alexa Fluor fluorescent donkey anti-rabbit/mouse secondary antibody (Thermo Fisher Scientific Inc., Massachusetts, USA) for an hour at room temperature.

## Results

A mouse pedigree designated MUTA-PED-C3PDE-242 (MPC-242) exhibiting early onset obesity and hyperinsulinemia was observed in a *N-ethyl-N-nitrosourea* (ENU) mutagenesis screen for age related disease (Potter et al. 2016). Briefly, ENU mutagenized C57BL/6 J male mice were crossed to C3H.Pde6b+ female mice to generate Generation-1 (G1) males who were then back-crossed to C3H.Pde6b+ females to generate a G2 population which was finally inter-crossed to generate a G3 population which was phenotyped for metabolic and other traits (Potter et al. 2016).

### Identification of the MPC-242 line and a mutation in *Pcsk1*

**Fig. 1:**
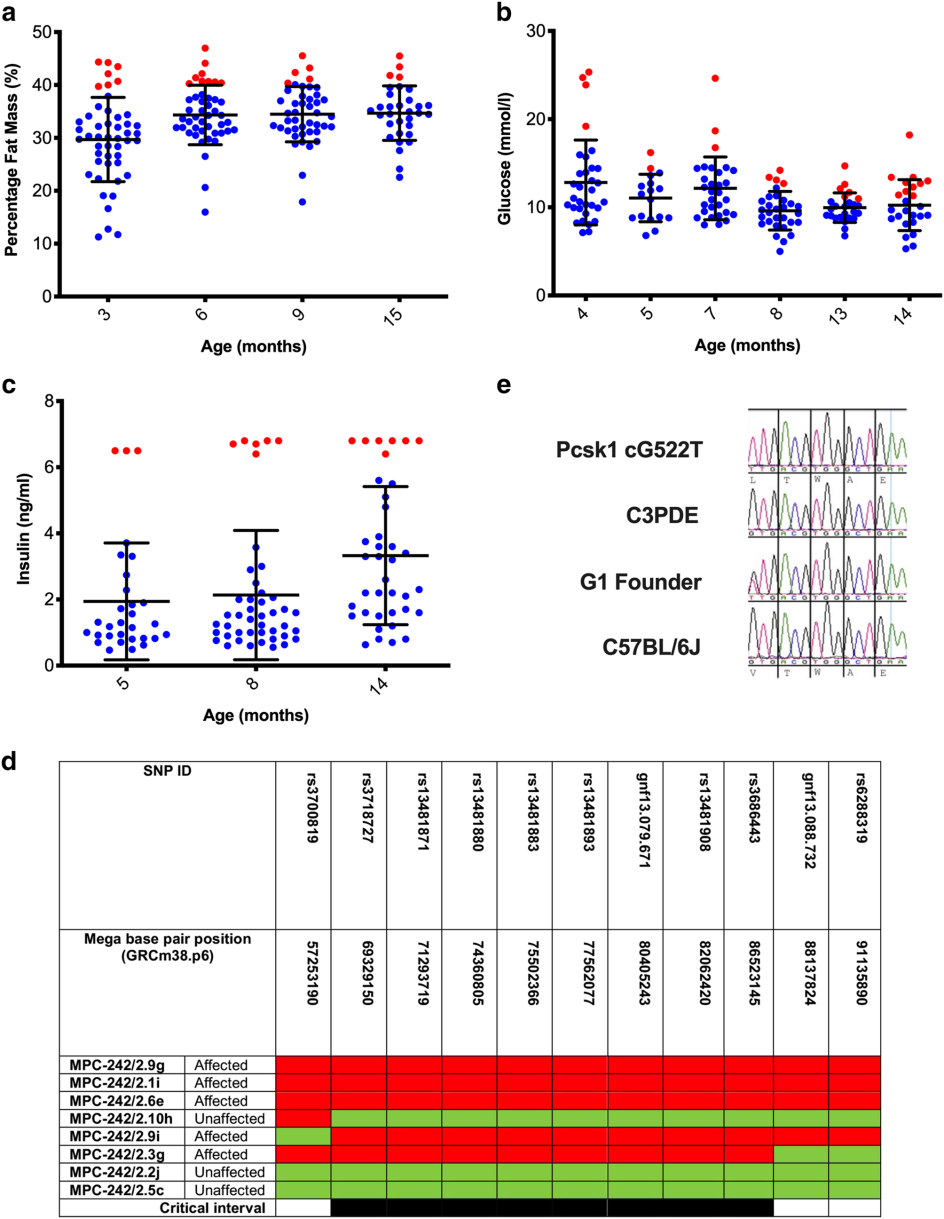
Identification of the MPC-242 line and a mutation in *Pcsk1*. **a** Male percentage fat mass at 3 (*n* = 48), 6 (*n* = 45), 9 (*n* = 44) and 15 (*n* = 32) months of age. **b** Male fasted plasma glucose concentration glucose at 3 (*n* = 32), 5 (*n* = 17), 7 (*n* = 30), 8 (*n* = 30), 13 (*n* = 25) and 14 (*n* = 25) months of age. **c** Male fasted plasma insulin concentration at 5 (*n* = 30), 18 (*n* = 44), and 14 (*n* = 38) months of age, note that the highest values are at the upper limit of the assay sensitivity. Mice more than 1 standard deviation from the mean are highlighted in red. Error bars are mean ± 1 standard deviation. **d** Mapping of the mutation in MPC-242 to a critical interval on mouse chromosome 13, using 3-month percentage fat mass and plasma insulin data from several age-points to identify affected mice. Red boxes mark homozygous C57BL/6 J DNA and green boxes heterozygous C57BL/6 J/C3H.Pde6b+ DNA. **e** sequence traces showing the mutation in *Pcsk1*, heterozygous in the founder G1 and homozygous in a G3 individual

MPC-242 was identified as containing individuals with elevated (greater than 1 standard deviation than the cohort population mean) percentage fat mass, plasma glucose and plasma insulin (Fig. 1a–c). Genotyping was carried out using the Illumina medium density single-nucleotide polymorphism panel of 1422 SNPs on five mice selected on their elevated 3-month fat mass plus 3 unaffected littermate controls. A common region of approximately 31 Mbp homozygous C57BL/6 J DNA was identified on chromosome 13 between rs3700819 and gnf13.088.732 (Fig. 1d). The G1 pedigree founder male and therefore an obligate carrier of the mutation was sequenced using next generation sequencing (NGS). Three putative coding mutations were identified, however only 1, cG522T corresponding to pV96L (Pcsk1-201 ENSMUST00000022075.5), in exon 3 of the proprotein convertase subtilisin/Keksin type 1 (*Pcsk1*) gene was confirmed by Sanger sequencing (Fig. 1e).

### Mis-splicing of exon 3 in ***Pcsk1***^***96L/96L***^ mice

**Fig. 2:**
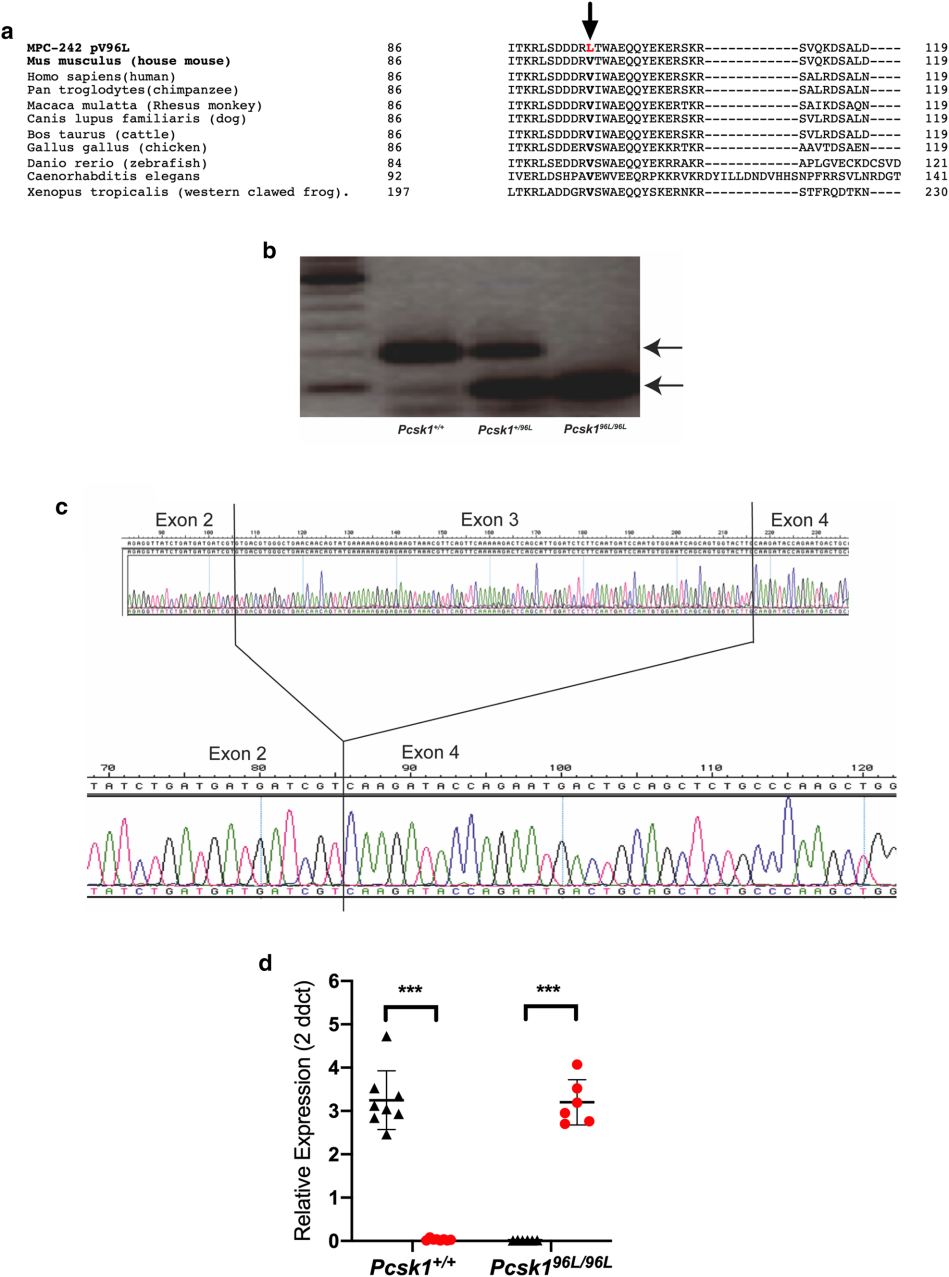
Mis-splicing of exon 3 in *Pcsk1*^*96L/96L*^ mice. **a** Protein sequence alignment of a segment of the *Pcsk1* gene carrying the V96L mutation (HomoloGene Multiple Alignment, NCBI). **b** PCR amplification of pancreas cDNA from wildtype, heterozygous and homozygous mutant mice using primers in exon 2 and 4 of *Pcsk1*. On the left is a 100 bp size ladder. Arrows indicate expected approximately 200 bp fragment and a shorter fragment indicative of exon 3 skipping, present weakly in wildtype, more strongly in heterozygotes and as the predominant fragment in homozygotes. **c** Sanger sequencing of the shorter PCR amplicons showing exon 3 skipping in cDNA amplified from homozygotes. **d** Quantitative PCR of wildtype and exon skipped transcripts. *Pcsk1*^+*/*+^ mice *Pcsk1*^*96L/96L*^ mice *n* = 8 and *n* = 8 at for the wildtype transcript, and *n* = 6 and *n* = 6 for the exon skipped transcript, respectively. Expression is normalised to a *Hprt1* reference transcript. Black triangles and red circles indicate the wildtype and exon skipped transcripts, respectively. Mean ± standard deviation, < 0.001 represented as ***. Differences in expression between the two transcripts in *Pcsk1*^+*/*+^ mice and *Pcsk1*^*96L/96L*^ mice were tested using an unpaired Mann–Whitney test

Sequence alignment showed that the valine residue at position 96 of *Pcsk1* is highly conserved and are predicted by SIFT [https://sift.bii.a-star.edu.sg (Sim et al. 2012)] and PolyPhen-2 [https://genetics.bwh.harvard.edu/pph2/index.shtml (Adzhubei et al. 2010)] to affect protein function (0.03) and be probably damaging (score 0.999), respectively (Fig. 2a). However, cG522T is also the first nucleotide of exon 3 and thus the mutation disrupts the splice acceptor site (….tttat**agGTGA**CG… to ….tttat**ag**T**TGA**CG… [where the exon is uppercase, the intron is lower case, the mutation is underlined and nucleotides are in bold for the consensus splice site bases)] and could therefore disrupt exon splicing. In order to test this, RNA was extracted from wildtype, heterozygous and homozygous mutant pancreas tissue and cDNA prepared and PCR amplified with primers in exon 2 and 4 (Fig. 2b). In wildtype cDNA, one band of the expected size was detected, in heterozygotes two bands, one of the expected size and one of a smaller size consistent with skipping exon 3, and in homozygotes only the latter band was detected (Fig. 2b). Sequencing of the fragments demonstrated that exon 3 was indeed being skipped in mutant mice (Fig. 2c). Finally, we quantified the two splice products (wildtype and mutant) in hypothalamic RNA using TaqMan quantitative RT-PCR. In homozygous mutant mice we found very low levels of wildtype transcript and high levels of exon 3 skipped transcript (Fig. 2d). The predominant transcript in wildtype mice was full length (Fig. 2d). Thus, the *Pcsk1*^*96L/96L*^ mutations is a strong hypomorph as there is some residual detectable wildtype spliced transcript.

Mice from the G3 generation were then repeatedly backcrossed to C3H.Pde6b+ mice to generate further backcross generations. Specific generations (G1–G12) were then inter-crossed to generate phenotyping cohorts.

### ***Pcsk1***^***96L/96L***^ mice are obese and showed reduced lean mass as a proportion of bodyweight

**Fig. 3:**
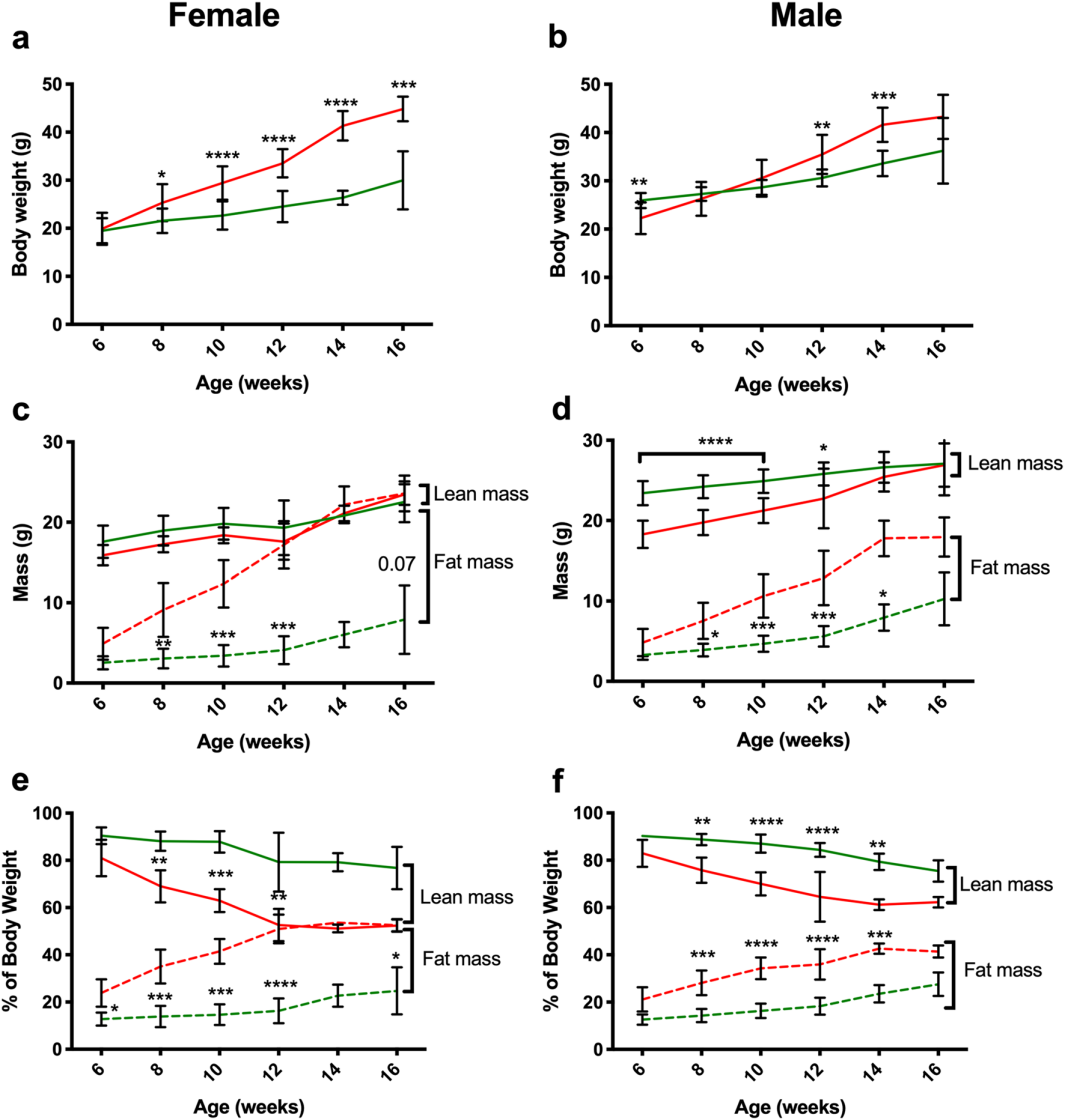
Obesity in G6 *Pcsk1*^*96L/96L*^ mice. **a**, **b** Increased body weight over 16 weeks in female and male mutant mice, respectively. **c**, **d** Increased fat mass over 16 weeks in female and male mutant mice, respectively, and reduced lean mass in male mutant mice. **e**, **f** Increased relative fat mass over 16 weeks in female and male mutant mice, respectively, and reduced lean mass in both male and female mutant mice. *Pcsk1*^+*/*+^ mice *Pcsk1*^*96L/96L*^ mice *n* = 14 and *n* = 9 at 6,8,10 and 12 weeks, and *n* = 4 and *n* = 4 at 14 weeks and *n* = 5 and *n* = 11 at 16 weeks, respectively. Green and red lines indicate *Pcsk1*^+*/*+^ mice and *Pcsk1*^*96L/96L*^ mice, respectively. Mean ± standard deviation, *p* =  < 0.05, < 0.01, < 0.001 and < 0.0001 represented as *, **, ***, and ****. **a**, **b** Body weight and **d** lean mass, analysed by multiple *t* test with Holm–Sidak correction for multiple testing. In the case of **a**, **b** data were log transformed before analysis, **c** lean and fat mass, **d** fat mass, analysed by 1-way ANOVA Kruskal–Wallis test with Dunn’s correction for multiple testing. **e**, **f** Percentage lean and fat mass, analysed by 1-way ANOVA Kruskal–Wallis test with Dunn’s correction for multiple testing

Mice at G6 were weighed every 2 weeks and body composition determined by quantitative NMR (EchoMRI). Both male and female homozygous *Pcsk1*^*96L/96L*^ mice showed increased body weight although in the case of males they were initially lighter at 6 weeks of age (Fig. 3a, b). These mice showed increased absolute fat mass from 8 weeks of age and in the case of males reduced lean mass over weeks 6–12 (Fig. 3c, d). As a proportion of body weight both males and females showed increased fat mass and reduced lean mass (Fig. 3e, f). These patterns of body composition change varied qualitatively across the generations (G3, G6 and G12) but followed a similar pattern (data not shown). *Pcsk1*^*96L/96L*^ mice also had a shorter body length by 1.05 ± 0.19 cm (Mann–Whitney test *p* ≤ 0.0001, 10 wildtype and 9 homozygous mice) than wildtype litter mates, as measured in G9 males at 16 weeks of age.

### ***Pcsk1***^***96L/96L***^ mice are hyperphagic

**Fig. 4:**
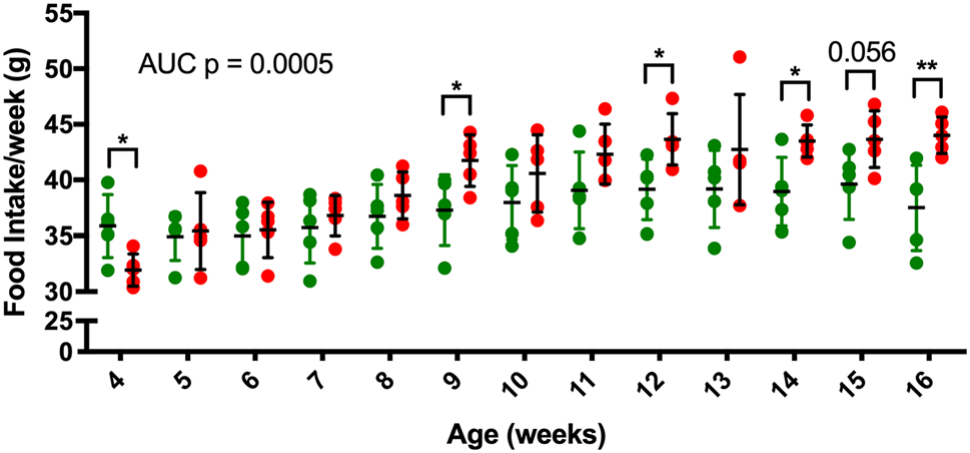
Increased food intake in G9 *Pcsk1*^*96L/96L*^ mice. Male mice were housed in pairs and food intake measured by weighing food daily. Food intake was totalled over a week and an estimated food intake by mouse calculated. *Pcsk1*^+*/*+^ mice *Pcsk1*^*96L/96L*^ mice *n* = 10 and 10 in pairs within 5 cages. Red and green filled circles are homozygous and wildtype mice, respectively. Mean ± standard deviation, *p* ≤ 0.05, < 0.01, < 0.001 and < 0.0001 represented as *, **, ***, and ****. AUC and individual time points were tested using an unpaired *t* test

In order to determine whether obesity in mutant mice was due to increased food intake, we paired G9 male mice from 4 weeks of age and measured weekly food intake up to 16 weeks of age (Fig. 4). The area under the food intake curves (AUC) over the 12-week measurement period was significantly increased for homozygous mutant mice (*p* = 0.0005). At 16 weeks, food intake was increased by 6.51 ± 1.633 g. Indicating increased energy intake over time likely explains the increased fat mass in these mice.

### *Pcsk1*^*96L/96L*^ mice are glucose intolerant and hyperproinsulinaemic.

**Fig. 5:**
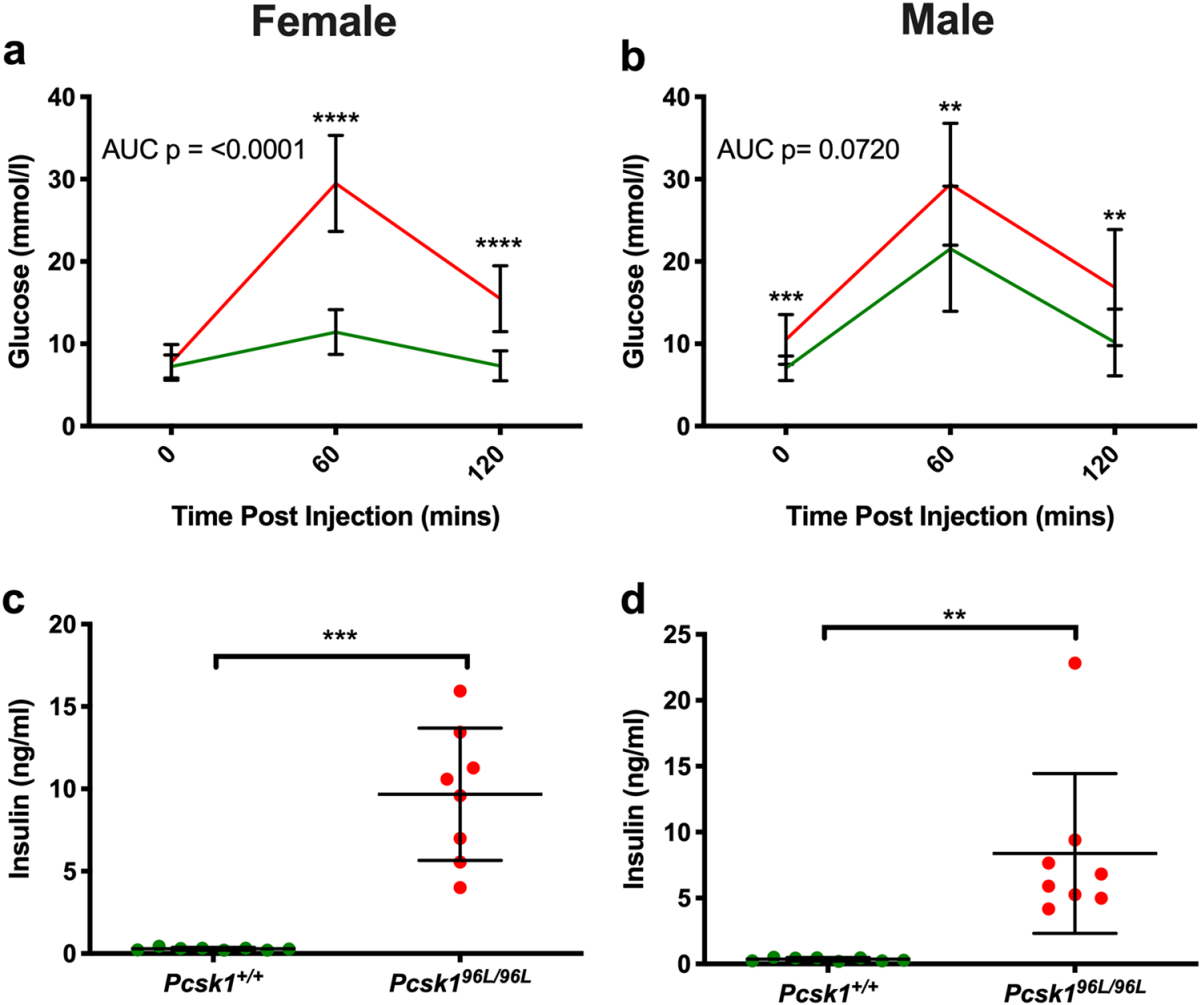
Impaired glucose tolerance and hyperinsulinemia in G6 *Pcsk1*^*96L/96L*^ mice. **a**, **b** Impaired glucose tolerance at 12 weeks of age in female and male mutant mice. *Pcsk1*^+*/*+^ mice and *Pcsk1*^*96L/96L*^ mice *n* = 13 and 12, and *n* = 8 and 18, female and male, respectively. **c**, **d** Hyperinsulinemia at 12 weeks of age in female and male mutant mice. *Pcsk1*^+*/*+^ mice and *Pcsk1*^*96L/96L*^ mice *n* = 8 and 12, and *n* = 8 and 8, female and male, respectively. **a** AUC and individual time points were tested using an unpaired Mann–Whitney test. **b** AUC was compared with an unpaired *t* test and then individual time points were tested using an unpaired Mann–Whitney test. Green and red lines or filled circles indicate *Pcsk1*^+*/*+^ mice *Pcsk1*^*96L/96L*^ mice, respectively. Mean ± standard deviation, *p* ≤ 0.05, < 0.01, < 0.001 and < 0.0001 represented as *, **, ***, and ****

**Fig. 6:**
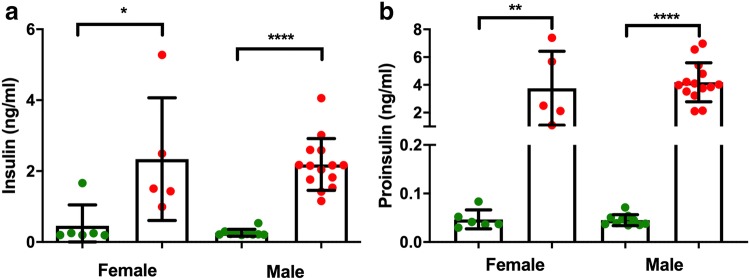
Hyperproinsulinaemia in *Pcsk1*^*96L/96L*^ mice. **a**, **b** Insulin and proinsulin concentrations determined using ELISA on overnight fasted plasma samples from female and male 12-week-old mice congenic on C3H.Pde6b+. *Pcsk1*^+*/*+^ mice and *Pcsk1*^*96L/96L*^ mice *n* = 6 and 5, and *n* = 10 and 14, female and male, respectively. **a**, **b** Unpaired two-tailed Mann–Whitney test. Green and red filled circles indicate *Pcsk1*^+*/*+^ mice and *Pcsk1*^*96L/96L*^ mice, respectively. Mean ± standard deviation, *p* ≤ 0.05, < 0.01 and < 0.0001 represented as *, ** and ****

Male G6 mice showed fasting hyperglycaemia and both males and females showed glucose intolerance (Fig. 5a, b). Given the role of PCSK1 in insulin processing, we then measured proinsulin and insulin in blood plasma from mice that were congenic on the C3H.Pde6b+ background. Both males and females showed fasting hyperinsulinaemia (Fig. 5c, d). However, the insulin ELISA assay is reported to also partially detect proinsulin if present. Therefore, in order to further investigate whether PCSK1 deficiency was leading to incomplete cleavage of insulin and thus secretion of proinsulin, we carried out a further ELISA assay specific for mouse proinsulin in the same samples from congenic mice (Fig. 6a, b). Homozygous mutant mice showed hyperproinsulinaemia and this elevated proinsulin may explain a proportion of the elevated insulin measurements. Insulin levels were apparently higher in G6 mice than congenic mice which may reflect changes in the genetic background.

### Transient diarrhoea

In G6 and G12 cohorts, we observed that some homozygous mice, but not wildtype littermates, exhibited diarrhoea. Diarrhoea was transient occurring between 5 and 8 weeks of age and was of 3- to 17-day duration (*n* = 11).

### Defective PCSK1^96L^ maturation and trafficking

**Fig. 7:**
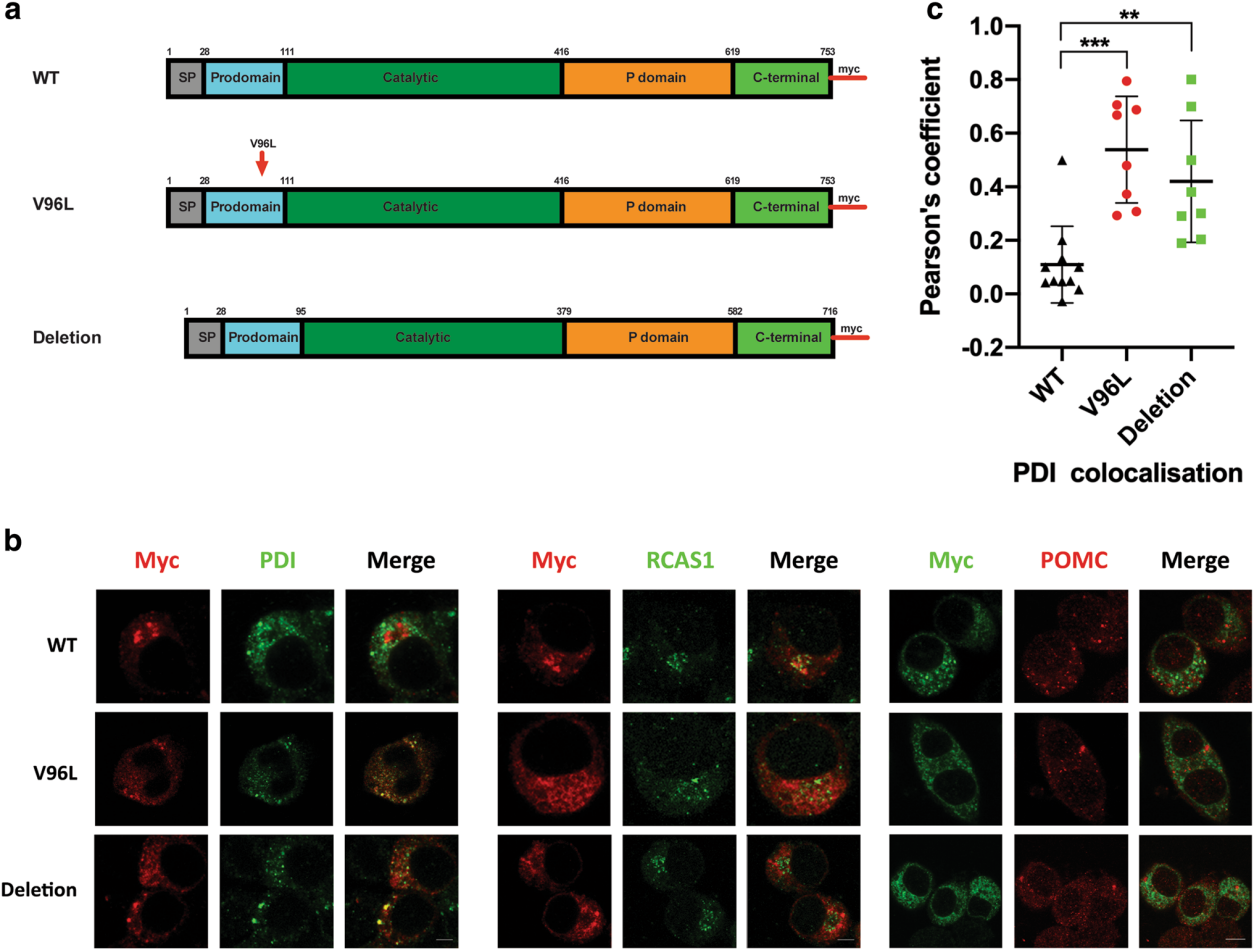
Mutant PCSK1 proteins are retained in the Endoplasmic Reticulum. **a** Diagram of the mouse PCSK1 protein indicating the position of V96L modifications. All three proteins have been myc tagged on the carboxy terminus. **b** Confocal images from Neuro2a cells transfected with the respective plasmids for 48 h and immunodetection of Myc, PDI or RCAS1 or POMC. **c** Pearson’s coefficient values of the colocalization of the three PCSK1 proteins with an ER marker PDI. Mean ± SD, **c** analysed by one-way ANOVA with Tukey’s multiple comparison test. **p ≤ 0.01, ***p ≤ 0.001. Scale bar 10uM

In order to investigate the effect of the *Pcsk1*^*96L*^ mutation we subcloned the mouse Pcsk1 cDNA into pcDNA^TM^3.1/*Myc*B vector under the control of a CMV promoter. Three sequence validated constructs were made, a wildtype, a 96L mutant and an exon 3 deletion (Fig. 7a). All three were C-terminal Myc tagged and carried two additional C-terminal modifications to prevent cleavage at the C-terminal and thus loss of the tag (Bernard et al. 2003). The constructs were then transiently transfected into mouse neuroblastoma Neuro2a cells for 48 h before co-immunostaining for the Myc tag and RCAS1 (Golgi marker) or PDI (Endoplasmic Reticulum marker) or POMC (neuropeptide vesicle marker) (Fig. 7b). The wildtype construct was distributed unevenly across the cell but was not colocalised to ER, Golgi or secretory vesicles. In contrast both 96L and the exon 3 deletion proteins were colocalised to some degree to the ER (Fig. 7c).

## Discussion

The *Pcsk1*^*96L/96L*^ mutation results in the substitution of a highly conserved amino acid which was predicted to have a damaging effect. However, as the mutated nucleotide is the first in exon 3, it also disrupted a splice acceptor site resulting in skipping of exon 3 and its loss from the transcript. The full-length transcript was consequently expressed at very low levels in mutant homozygotes. Similar to our model, the exon 1 deletion of Zhu et al. showed hyperproinsulinaemia in addition to multiple neuroendocrine peptide processing defects (Zhu et al. 2002b). However, in contrast to our model, exon 1 deletion mice were not obese and exhibited dwarfism possibly due to defects in hypothalamic growth hormone-releasing hormone (GHRH) (Zhu et al. 2002b). However, consistent with a possible growth hormone processing defect, our p96L mice were significantly shorter than their wildtype littermates.

Our model also differs from the Mbikay et al*.* 32.7 kb deletion of exon 3 to 9, with insertion of PGKneo, mouse which showed homozygous preimplantation lethality (Mbikay et al. 2007). The severity of this 32.7 kb deletion allele may be due to the expression of aberrant gene proteins which may inhibit other proprotein convertases, in a dominant-negative fashion, which may otherwise have partially compensated for *Pcsk1* deficiencies (Mbikay et al. 2007). Our pV96L mouse model is most similar, in causing hyperproinsulinaemia, impaired glucose tolerance, hyperphagia and obesity, to the N222D *Pcsk1* mouse model described by Lloyd et al. (2006). Hyperphagia in this model may in part be due to defective POMC processing and perturbation of the melanocortin pathway in satiety, and this may also be part of the mechanism in our model, given that we also observed hyperphagia (Lloyd et al. 2006; Stijnen et al. 2016).

A summary of these phenotypic comparisons between our model and those published is provided in Table 1.

**Table 1:** Phenotypic comparison of the published mouse models of *Pcsk1* deficiency

Mutation	Genotype	Diarrhoea	Obesity	Hyperphagia	Growth alterations	Impaired glucose tolerance	Prohormone processing defects/cytokine alterations				
							Proglucagon	Proinsulin	POMC	Pro-GHRH	Macrophage cytokine
Exon 1 deletion^a^	Homozygous Reduced viability (~ 1 third surviving beyond 7 days)^a^	Chronic mild^a^	No^a^ Based on weight		Dwarfism (60% of normal weight at 6 wks)^a^	No^a^	Yes^a^ Blocked intestinal GLP-1/-2 production	Yes^a^ Pancreas extracts	Yes^a^ Pituitary ACTH deficiency	Yes^a^ Very low GHRH in hypo-thalamic extracts	Altered Cytokine secretion^b^
	Heterozygous		Yes, mild^a^ Based on weight			Mild^a^					
Exons 3–9 deletion^c^	Homozygous—complete preimplantation lethality^c^										
	Heterozygous		Mildly increased weight for females on high-fat diet at 17 wks^c*^		Reduced weight in females on a low-fat diet at 17 wks^c*^				No, MS peptide profiles in anterior pituitary similar^c^		
N222D amino acid substitution^d^	Homozygous (no lethality reported)		Yes^d^ Due to fat mass	Yes^d^	No^d^	Yes^d^		Yes^d,e^ Isolated islets	Yes 45% reduced hypothalamic MSH^d^ In contrast, *increased* plasma ACTH^d^		
	Heterozygous		Yes, mild^d^ Due to fat mass								
V96L amino acid substitution^f^	Homozygous Viable	Transient^f^	Yes^f^ Due to fat mass	Yes^f^	Mildly reduced length^f^	Yes^f^		Yes^f^ Plasma			

Superscript numbers refer to publication references ^a^(Zhu et al. 2002a), ^b^(Refaie et al. 2012), ^c^(Mbikay et al. 2007), ^d^(Lloyd et al. 2006), ^e^(Prabhu et al. 2014) and ^f^(Muhsin et al. this manuscript). Note that the *Pcsk1* deletion studies did not measure body composition, only weight, which may miss changes in the proportions of fat and lean mass. *, this study indicates that mice were fed for 13 weeks on each diet and that no effect was found in males (Mbikay et al. 2007). Comparisons are with wildtype mice

*POMC* proopiomelanocortin, *ACTH* adrenocorticotropic hormone, *pro-GHRH* pro-growth hormone-releasing hormone, *GLP-1/-2* glucagon like peptide 1 and 2, *wks* weeks of age, *PGKneo* phosphoglycerate kinase promoter driven neomycin resistance gene, *MSH* melanocyte-stimulating hormone, *MS* mass spectrometry analysis

Loss of exon 3 in the cG522T/pV96L allele removes the primary autocatalytic propeptide cleavage site between the propeptide and catalytic domain (Boudreault et al. 1998; Rabah et al. 2006; Stijnen et al. 2016). The propeptide domain is necessary for protease folding in the ER and once cleaved the protein is exported to the Golgi, although the cleaved peptide remains associated as an autoinhibitor (Rabah et al. 2006; Stijnen et al. 2016).This propetide is subsequently disassociated after a second site cleavage, leading to active PCSK1. Further COOH-terminal cleavage occurs in secretory vesicles (Stijnen et al. 2016). We found that overexpression in Neuro2a cells of a tagged exon 3 deletion or the 96L mutant protein resulted in its retention in the ER, in contrast to wildtype protein (all three constructs were modified to prevent C-terminal cleavage of the Myc Tag). The mutant proteins are therefore not processed properly which is necessary for activation. Similarly, the N222D mutant is also reported to show predominant ER retention in transfected Rin5f and Neuro2a cells as well as increased susceptibility to proteosomal degradation, and low levels of secreted enzyme (Prabhu et al. 2014). Further, Blanco et al*.* have reported a 90% lower enzymatic activity in secreted N222D mutant protein transfected into HEK293 cells, although in these experiments, protein was secreted at similar levels to wildtype and there was also some colocalization with a secretory granule marker (Blanco et al. 2015). From these data it is unclear why the exon 1 knockout mouse of Zhu et al*.* is different in terms of obesity compared to the N222D and our V96L model, although the latter models may be hypomorphic rather than nulls and the former exhibited a severe growth defect that may have obscured changes in adiposity. The different models may have differential effects on some but not all prohormone processing (Lloyd et al. 2006).

Both male and female homozygous 96L mice exhibit glucose intolerance and were clearly hyperproinsulinaemic. As proinsulin is only 3–5% as potent as insulin in binding to insulin receptors, this may account for the glucose intolerance (Freychet 1974).

One of the most common clinical presentations of *PCSK1* deficiency is severe malabsorptive diarrhoea diagnosed in the first 3 months of life which spontaneously improves after the age of 2 [reviewed (Stijnen et al. 2016)]. In two of the cohorts at G6 and G12, we observed as part of welfare monitoring transient diarrhoea in individual homozygous mice between 6 and 8 weeks of age, further suggesting that our model is a good phenocopy of the human disease.

In conclusion we report a second mouse model that exhibits some traits consistent with human patients carrying *PCSK1* mutations and that may therefore be useful in future studies of prohormone processing.
